# Liver-expressed antimicrobial peptide 2 antagonizes the effect of ghrelin in rodents

**DOI:** 10.1530/JOE-19-0102

**Published:** 2019-09-19

**Authors:** Md Nurul Islam, Yuichiro Mita, Keisuke Maruyama, Ryota Tanida, Weidong Zhang, Hideyuki Sakoda, Masamitsu Nakazato

**Affiliations:** 1 Division of Neurology, Respirology, Endocrinology and Metabolism, Department of Internal Medicine, Faculty of Medicine, University of Miyazaki, Miyazaki, Japan; 2 Systems Life Sciences Laboratory, Department of Medical Life Systems, Faculty of Life and Medical Sciences, Doshisha University, Kyoto, Japan; 3 Department of Sports and Fitness, Faculty of Wellness, Shigakkan University, Aichi, Japan; 4 CREST (Japan) Agency for Medical Research and Development (A-MED) 1-7-1 Otemachi, Tokyo, Japan

**Keywords:** ghrelin, LEAP2, GHSR, food intake, blood glucose, growth hormone

## Abstract

Ghrelin, a stomach-derived peptide, promotes feeding and growth hormone (GH) secretion. A recent study identified liver-expressed antimicrobial peptide 2 (LEAP2) as an endogenous inhibitor of ghrelin-induced GH secretion, but the effect of LEAP2 in the brain remained unknown. In this study, we showed that intracerebroventricular (i.c.v.) administration of LEAP2 to rats suppressed central ghrelin functions including Fos expression in the hypothalamic nuclei, promotion of food intake, blood glucose elevation, and body temperature reduction. LEAP2 did not inhibit neuropeptide Y (NPY)-induced food intake or des-acyl ghrelin-induced reduction in body temperature, indicating that the inhibitory effects of LEAP2 were specific for GHSR. Plasma LEAP2 levels varied according to feeding status and seemed to be dependent on the hepatic *Leap2* expression. Furthermore, ghrelin suppressed the expression of hepatic *Leap2* via AMPK activation. Together, these results reveal that LEAP2 inhibits central ghrelin functions and crosstalk between liver and stomach.

## Introduction

Ghrelin, a 28-amino acid acylated peptide originally isolated from rat and human stomach, is involved in the regulation of energy homeostasis and growth hormone (GH) secretion (Kojima *et al.* 1999, Nakazato *et al.* 2001). The ghrelin signal is communicated to the hypothalamus, the center of regulation of feeding behavior, via the vagal afferent nerve (Yanagi *et al.* 2018). The activities of ghrelin are mediated through binding to the growth hormone secretagogue receptor (GHSR), a G-protein-coupled receptor (GPCR). GHSR is expressed at high levels in the hypothalamic arcuate nucleus (ARC), dorsomedial hypothalamus (DMH), ventromedial hypothalamic nucleus (VMH), and lateral hypothalamic nucleus (LH) (Mitchell *et al.* 2001, Zigman *et al.* 2006).

Liver-expressed antimicrobial peptide 2 (LEAP2), a 40 amino acid peptide, was originally isolated by comprehensive chromatographic characterization of human hemofiltrate (Krause *et al.* 2003). Human LEAP2 is processed from a 77 amino acid precursor by a furin-like endoprotease, and its amino acid sequence is identical to that of murine LEAP2 (Krause *et al.* 2003). Like many antimicrobial peptides, LEAP2 has two disulfide bonds. LEAP2 is the second blood-derived peptide to be discovered that is predominantly expressed in the liver and exhibits antimicrobial activity; LEAP1/hepcidin was the first (Krause *et al.* 2000). In high fat diet-induced obese mice, vertical sleeve gastrectomy, a common surgery for the treatment of obesity, resulted in increased expression of *Leap2* in the stomach and decreased expression in the duodenum (Ge *et al.* 2018). LEAP2 was tested against 168 known GPCRs using PathHunter beta-arrestin enzyme fragment complementation technology (Ge *et al.* 2018). LEAP2 directly interacted with GHSR and inhibited GHSR activation via a noncompetitive mechanism (Ge *et al.* 2018). The sequence of the LEAP2 peptide is highly conserved across mammals, implying that control of GHSR by LEAP2 is a general mechanism.

Both central and peripheral administration of ghrelin induced food intake in multiple species, including humans (Nakazato *et al.* 2001, Wren *et al.* 2001), whereas systemic administration of LEAP2 to mice suppressed ghrelin-induced food intake and GH release (Ge *et al.* 2018). Therefore, we performed intracerebroventricular (i.c.v.) administration of LEAP2 in rats, and examined its suppressive effects on ghrelin-induced changes in food intake, blood glucose level, and body temperature. We also monitored plasma LEAP2 level and liver *Leap2* expression under *ad libitum,* fasting, and re-feeding conditions. Finally, we sought to identify the mechanism of *Leap2* expression in the liver.

## Materials and methods

### Animals

Eight-week-old male Wistar rats (Charles River Laboratories), weighing 280–300 g, and 12-week-old male C57BL/6J mice, weighing around 25 g (Charles River Laboratories) were housed in an animal center and maintained under a 12-h/12-h light/darkness cycle (light on 08:00 and off at 20:00) at constant temperature (23 ± 1°C). Rats were fed a regular chow diet (CLEA Rodent Diet CE-2, CLEA Japan, Tokyo, Japan) and allowed free access to water. For i.c.v. operations, rats were anesthetized by i.p. injection of a mixture of three anesthetics (Domitor, 1 mg/mL; midazole, 5 mg/mL; vetorphale, 5 mg/mL) in a volume of 1.5 mL/300 g body weight and mounted on a stereotaxic apparatus; then, a commercial stainless steel guide cannula was implanted into the left lateral ventricle (8.0 mm anterior from the interaural line, 1.5 mm lateral from the sagittal suture, and 3.0 mm ventral from the dura), according to the Rat Brain Atlas (Paxinos and Watson, Fourth Edition). After the operation, the rats were placed in individual cages and allowed to recover for at least 7 days, during which they were handled daily for acclimation. Ghrelin (human), LEAP2 (human), des-acyl ghrelin (rat), NPY, and GLP-1 were purchased from Peptide Institute (Osaka, Japan). [D-Lys^3^]-GHRP-6, an antagonist for GHSR, was purchased from Tocris Bioscience. i.c.v. injection was carried out with an injector cannula (27G, 1 mm long from the tip of the guide cannula). Mice were anesthetized after overnight (20:00–08:00) fasting, and ghrelin was introduced through the inferior vena cava. Two hours after ghrelin administration, plasma and tissue samples were collected for LEAP2 analysis. 38-week-old GHSR-null mice (Zigman *et al.* 2005) which harbor a loxP-flanked transcriptional blocking cassette in both endogenous GHSR alleles were kindly provided by Dr Syu Takeda at Tokyo Medical and Dental University. In comparing study between wild-type (WT) and GHSR-null mice, 15-week-old male WT and 19-week-old GHSR-null mice were used. All animal experiments were approved by the Animal Care and Use Committee of University of Miyazaki.

### Peptide dose

For i.c.v. injection, doses of peptides were as follows: ghrelin, 0.1 nmol; des-acyl ghrelin, 0.5 nmol; NPY, 1.2 nmol; GLP-1, 0.3 nmol; LEAP2, 1 nmol; and [D-Lys3]-GHRP-6, 20 nmol. Peptides for i.c.v. injection were dissolved in 5 µl of artificial cerebrospinal fluid (aCSF) per rat. For i.p. injection, the peptide doses (otherwise stated) were as follows: ghrelin, 5 nmol; LEAP2, 15 nmol. These peptides were dissolved in 0.5 mL of saline per rat. For mouse experiments, ghrelin was administered intravenously (i.v.) at a dose of 60 nmol/kg body weight. For hepatocytes, peptide doses were as follows: ghrelin, 10 nM; des-acyl ghrelin, 10 nM in Dulbecco’s Modified Eagle Medium (DMEM).

### Feeding experiment

Single i.c.v. injections of ghrelin, ghrelin + LEAP2, NPY, or NPY + LEAP2 were carried out at 09:45 h, and pre-weighed foods were supplied at 10:00 h to measure food intake at that time point. To measure dark-phase food intake, rats were deprived of food from 16:00 h to 19:45 h. Following i.c.v. injection of either LEAP2 or aCSF, rats were supplied with food at 20:00 h, and subsequent food intake was measured at several time points. To measure fasting-induced food intake, rats were kept on a fast from 18:00 h to 08:00 h; following i.c.v. injection, 2-h food intake was measured. In the case of peripheral administration, LEAP2 was administered at 09:30 h, and ghrelin 0.5 h later, after which 1-h and 2-h food intake were measured.

### Fos immunohistochemistry

Rats (*n* = 3/group) received an i.c.v. injection of ghrelin or ghrelin + LEAP2. After 90 min, they were anesthetized with a mixture of three anesthetics (Domitor, 1 mg/mL; midazole, 5 mg/mL; vetorphale, 5 mg/mL) in a volume of 0.1 mL/20 g body weight and transcardially perfused with ice-cold PBS and ice-cold 4% PFA. Hypothalamic sections (thickness, 40 µm) were cut on a freezing microtome (Yamato Kohki Industrial, Saitama, Japan). For Fos immunohistochemistry, free-floating sections were blocked in blocking buffer (3% normal serum in 0.3% PBS-T) for 60 min, and then incubated overnight at 4°C with rabbit anti-c-Fos (Santa Cruz Biotechnology). After washing with PBS, the sections were incubated for 60 min at room temperature with Alexa Fluor 488-labeled anti-rabbit IgG antibody (1:500; Invitrogen). Images were visualized by confocal microscopy. Fos-positive cells were counted manually.

### Blood glucose and body temperature measurement

Ghrelin or ghrelin + LEAP2 was administered i.c.v. to conscious rats at 16:00 h. After injection, blood glucose level was monitored using a glucometer (Terumo, Tokyo, Japan). Food and water were removed during the blood glucose experiment. For measurement of body temperature, rats were anesthetized by i.p. injection of urethane. i.c.v. injections were given at 16:00 h, and rectal temperature was measured with a digital thermometer (TD-300; Shibaura Electronics, Saitama, Japan).

### Measurements of plasma LEAP2, ghrelin, and insulin

Blood samples were taken from anesthetized rats by cardiac puncture and collected in a tube containing aprotinin and EDTA (Wako). Samples were immediately centrifuged at 4°C, and plasma was collected. ELISA for plasma LEAP2 was developed as previously described (Mita *et al.* 2017). Briefly, plasma was processed using a 10-kDa column (AmiconUltra 0.5 mL Centrifugation Filters; Merck Millipore), and the filtrate was subsequently used for measurement of the LEAP2 concentration. Next, 96-well microtiter plates were coated with LEAP2 standard peptide or plasma in 100 mM sodium bicarbonate buffer (pH 9.6) and incubated overnight at 4°C. After washing with washing buffer (PBS containg 0.05% Tween-20), non-specific binding was blocked with 1% BSA in PBS for 1 h at 37°C. For detection of plasma LEAP2, rabbit polyclonal anti-LEAP2 antibody (1:3200; H075-40, Phoenix Pharmaceuticals, Burlingame, CA, USA) was added and incubated at 37°C for 1 h. After washing in washing buffer, HRP-linked anti-rabbit IgG (#7074; Cell Signaling Technology) in washing buffer containing 0.1% BSA was added, and the sample was incubated for 1 h at 37°C. Finally, the plate was washed five times and TMB was added. The enzyme–substrate reaction was allowed to proceed for 5–10 min at room temperature under dark environment. The reaction was stopped by the addition of 1 N H_2_SO_4_, and absorbance was immediately read at 450 nm in a 96-well microplate reader (iMark^TM^ microplate absorbance reader; Bio-Rad). Data demonstrating the verification of this ELISA system are provided in Supplementary Table 1 (see section on supplementary materials given at the end of this article). The inter-assay precision coefficient of variation was <5%, assay range 12–1000 ng/mL. Plasma insulin was measured using a rat insulin EIA kit (Morinaga Institute of Biological Science, Yokohama, Japan). The plasma ghrelin assay was a two-site immuno-enzymatic assay requiring 100 µL of plasma, performed automatically on an AIA-600II immunoassay analyzer (Tosoh, Tokyo, Japan).

### Measurement of GH release

Primary cells were isolated from rat pituitary as previously described (Kojima *et al.* 1999). Primary pituitary cells were stimulated with LEAP2 (1 µM) and/or ghrelin (100 nM). To examine the effect of LEAP2 on GH release *in vivo*, saline or LEAP2 (15 nmol in 50 µL saline) was introduced through the jugular vein. After 10 min, ghrelin (5 nmol) was administered through the jugular vein, and blood samples were taken from the tail tip at 0, 15, and 30 min. GH was measured using the Rat/Mouse GH ELISA Kit (Millipore EMD).

### Extraction of mRNA and quantitative RT-PCR (qRT-PCR)

Total mRNA from all indicated tissues was extracted using the RiboPure Kit (Life Technologies Japan). First-strand cDNA was generated by reverse transcription using the High-Capacity RNA-to-cDNA Kit (Life Technologies). qRT-PCR was performed on a Thermal Cycler Dice Real Time System II (TaKaRa Bio) using SYBR Premix Ex Taq (2X) (TaKaRa Bio) and the following primer pairs: rat *Leap2*, 5′-AAAGACGACGCTGTTCCCTG-3′ and 5′-GAGCACTGTTGGAGGTGACTT-3′; mouse *Leap2*, 5′-GACCCCATTTTGGAGAGGGG-3′ and 5′-GGGAACAGCGTCTTTTTCTGC-3′; rat *Gapdh* 5′-GGCACAGTCAAGGCTGAGAATG-3′ and 5′-ATGGTGGTGAAGACGCCAGTA-3′; mouse *Gapdh* 5′-TGTGTCCGTCGTGGATCTGA-3′ and 5′-TTGCTGTTGAAGTCGCAGGAG-3′.

Three-dimensional digital PCR was performed on a ProFlex PCR System (Applied Biosystems) using the primer set for rat *Leap2* (Rn03648192_m1).

### Cell culture

Hepa1-6 cells were obtained from the American Type Culture Collection (Manassas). Hepa1-6 cells were maintained in DMEM containing 10% heat-inactivated FBS and antibiotics (100 U/mL penicillin, 100 μg/mL streptomycin). Cells were cultured at 37°C under an atmosphere of 95% air and 5% CO_2_.

Isolation of primary hepatocytes was described previously (Ariyoshi *et al.* 2017). Briefly, bone marrow cells were cultured in 1 mL Hepatocyte Culture Media (Lonza Walkersville, Walkersville, MD, USA) supplemented with 5% FBS for 6 h in a 37°C incubator with 5% CO_2_. One part conditioned medium was added to four parts of liver cell culture. Livers were minced to the level of 2 mm^3^. The pieces of liver were incubated for 30 min at 37°C with collagenase-Yakult (100 U/mL, Yakult Pharmaceutical, Tokyo, Japan). After collagenase treatment, the supernatant was decanted, and 5 mL of HGM supplemented with 10% FBS was added; the cells were then shaken at 160 rpm for 30 min at room temperature. After centrifugation at 50 × ***g*** for 2 min, the supernatant was removed, and 5 mL fresh HGM was added.

### Western blotting

Samples were lysed in RIPA buffer (Nacalai Tesque) containing phosphatase inhibitor (PhosSTOP, Roche). For detection of AMPKα (#2532, Cell Signaling Technology), phosphorylated AMPKα (Thr172) (#2535, Cell Signaling Technology), and β-actin (A2103, Sigma), equal amounts of protein were fractionated on Tris-glycine SDS-polyacrylamide gels, subjected to electrophoresis, and transferred to a PVDF membrane (Bio-Rad). Chemiluminescence was detected using the ECL Western blot detection kit (Wako).

### Statistical analysis

Student’s *t*-test was used to compare two groups, and one-way ANOVA was performed for multiple comparisons. All data are presented as means ± s.e.m. *P* < 0.05 was considered to indicate significance.

## Results

### Ghrelin regulates Leap2 mRNA expression in liver

**Figure 1:**
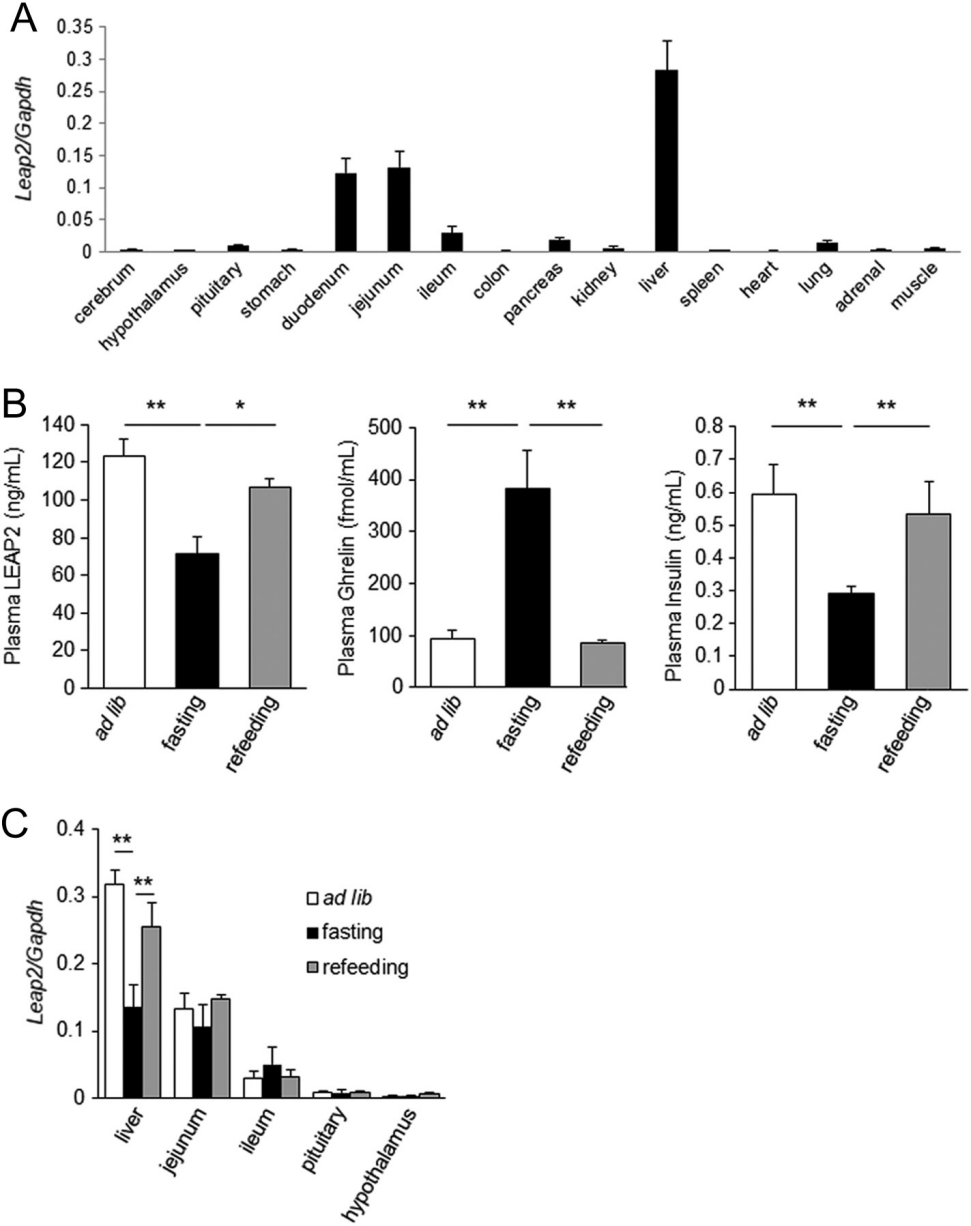
LEAP2 tissue distribution in rats. (A) *Leap2* was highly expressed in liver and small intestine, as determined by qRT-PCR (*n* = 4). (B) Plasma LEAP2, ghrelin, and insulin levels under the indicated feeding conditions (*n* = 8) (C) *Leap2* expression in liver was significantly decreased by fasting but returned to the basal level following re-feeding (*n* = 8). Bars represent means ± s.e.m. *P < 0.05, **P < 0.01.

*Leap2* mRNA was highly expressed in rat liver, as determined by qRT-PCR (Fig. 1A) and 3D digital PCR (Supplementary Fig. 1A). In mice, the *Leap2* mRNA level was highest in the jejunum (Supplementary Fig. 1B), as reported previously (Ge *et al.* 2018). Plasma levels of LEAP2, ghrelin, and insulin were measured under various feeding conditions (Fig. 1B). Following fasting, the plasma LEAP2 level decreased, but returned to the basal level after 6-h re-feeding. Insulin exhibited a similar pattern, whereas ghrelin behaved in the opposite manner; ghrelin levels increased with fasting and dropped after re-feeding. *Leap2* mRNA expression in the liver was significantly decreased by fasting, and increased by 6 h re-feeding (Fig. 1C). By contrast, *Leap2* mRNA expression did not alter in other organs (Fig. 1C).

**Figure 2:**
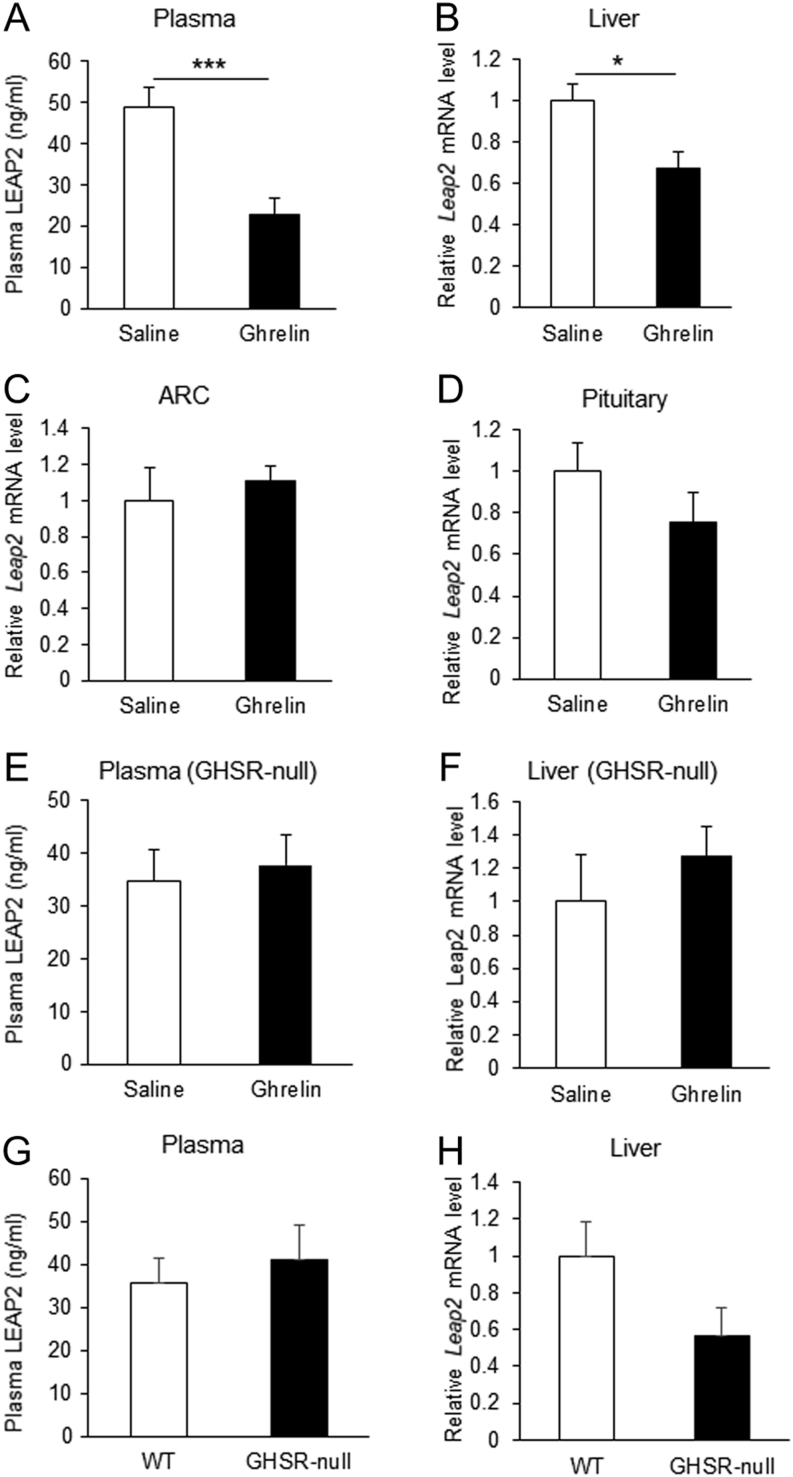
Ghrelin regulates LEAP2 expression in liver. i.v. administration of ghrelin to overnight-fasted mice caused a significant reduction in both plasma LEAP2 (A) and *Leap2* mRNA expression in liver (B), but not in hypothalamic ARC (C) or pituitary (D) (*n* = 8 for plasma, liver and ARC, *n* = 4 for pituitary). In GHSR-null mice, i.v. ghrelin did not change plasma LEAP2 (E) or *Leap2* mRNA expression in liver (F) (*n* = 3). Comparative analysis of plasma LEAP2 (G) and liver *Leap2* mRNA expression (H) in WT and GHSR-null mice (*n* = 3–6). Bars represent means ± s.e.m. *P < 0.05, ***P < 0.001.

**Figure 3:**
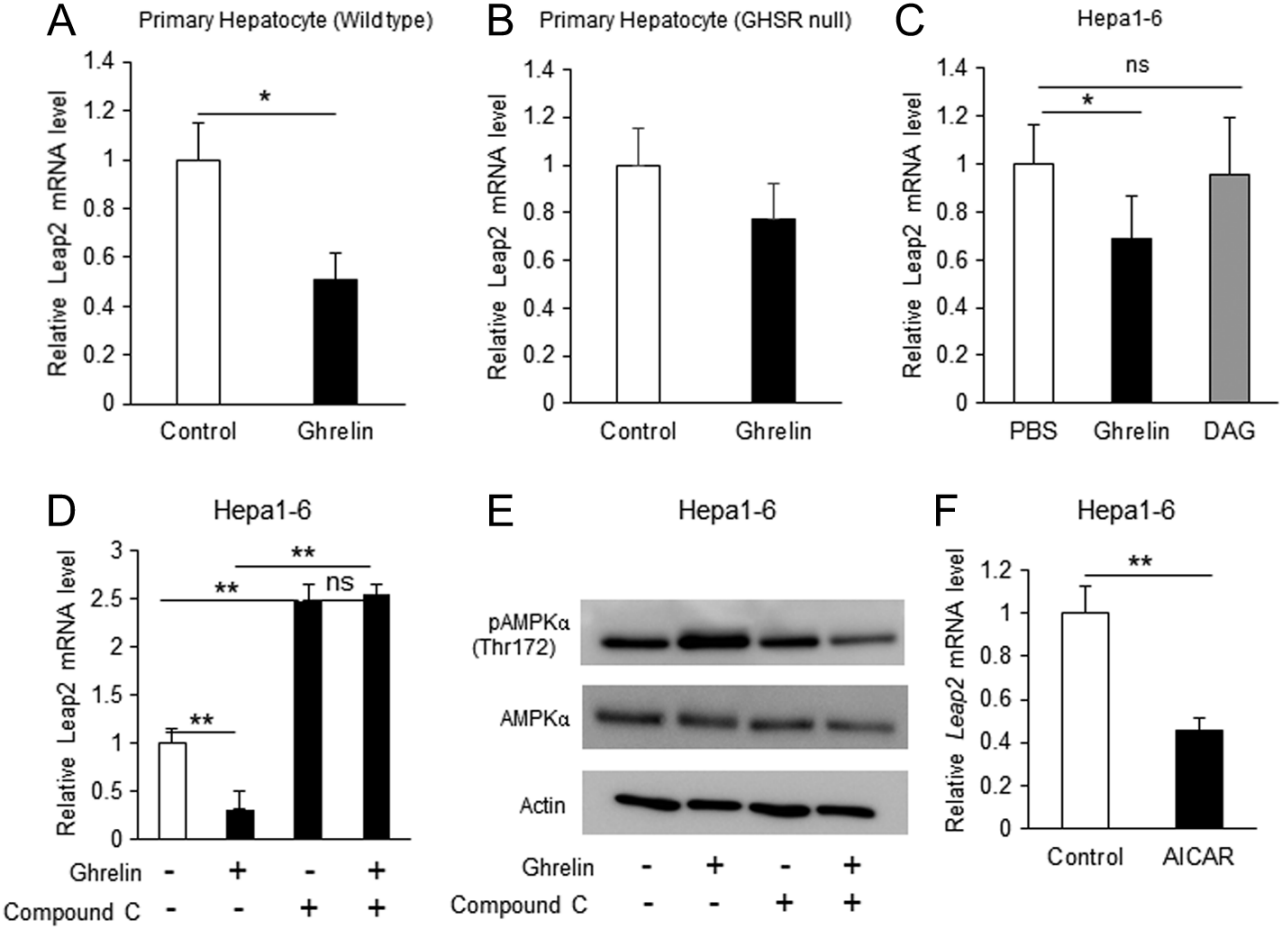
Ghrelin induces suppression of Leap2 expression through an AMPK-dependent pathway. (A) Ghrelin stimulation for 6 h decreased *Leap2* mRNA in primary hepatocytes of WT mice (*n* = 5), but (B) not in primary hepatocyte of GHSR-null mice (*n* = 5). (C) In Hepa 1–6 cells, ghrelin but not des-acyl ghrelin (DAG) suppressed *Leap2* mRNA expression (*n* = 5). (D) Compound C, an AMPK inhibitor, induced *Leap2* mRNA and abolished ghrelin-induced suppression of *Leap2* mRNA in Hepa 1–6 cells (*n* = 5). (E) Western blot analysis showing the impact of ghrelin on phosphorylated AMPKα (pAMPKα) (*n* = 3). (F) *Leap2* mRNA expression was decreased by AICAR, an AMPK activator (*n* = 5). Bars represent means ± s.d. or s.e.m. *P < 0.05, **P < 0.01**.

Next, we investigated whether ghrelin reduced *Leap2* mRNA expression in the liver. i.v. administration of ghrelin to fasted mice significantly decreased the levels of plasma LEAP2 (Fig. 2A) and *Leap2* mRNA in liver (Fig. 2B). However, i.v. ghrelin did not suppress *Leap2* mRNA expression in hypothalamic ARC (Fig. 2C) or pituitary (Fig. 2D). In GHSR-null mice, i.v. ghrelin did not suppress plasma LEAP2 (Fig. 2E) or *Leap2* mRNA level in the liver (Fig. 2F). The plasma LEAP2 level in WT and GHSR-null mice was not changed (Fig. 2G), whereas *Leap2* mRNA level in the liver of GHSR-null mice showed the tendency to decrease but did not reach the significant level compared to WT mice (Fig. 2H) after a 12-h fast. Ghrelin decreased *Leap2* mRNA in both primary mouse hepatocytes (Fig. 3A) and Hepa 1-6 cells (Fig. 3C). However, des-acyl ghrelin did not suppress *Leap2* mRNA in Hepa 1-6 cells (Fig. 3C) and ghrelin-induced suppression of *Leap2* expression was not significant in primary hepatocyte of GHSR-null mice (Fig. 3B). qRT-PCR cycle number for *Ghsr* mRNA was 33.2 ± 0.2 in primary mouse hepatocytes and 29.5 ± 0.4 in Hepa 1-6 cells. Next, we explored the pathway by which ghrelin suppresses *Leap2* expression. An AMPK inhibitor compound C abolished ghrelin-induced suppression of *Leap2* expression (Fig. 3D) and ghrelin-stimulated phosphorylation of AMPK (Fig. 3E) in Hepa 1-6 cells. *Leap2* mRNA in Hepa 1-6 cells was decreased by AICAR, an AMPK activator (Fig. 3F).

### Central LEAP2 suppresses ghrelin-induced feeding

**Figure 4:**
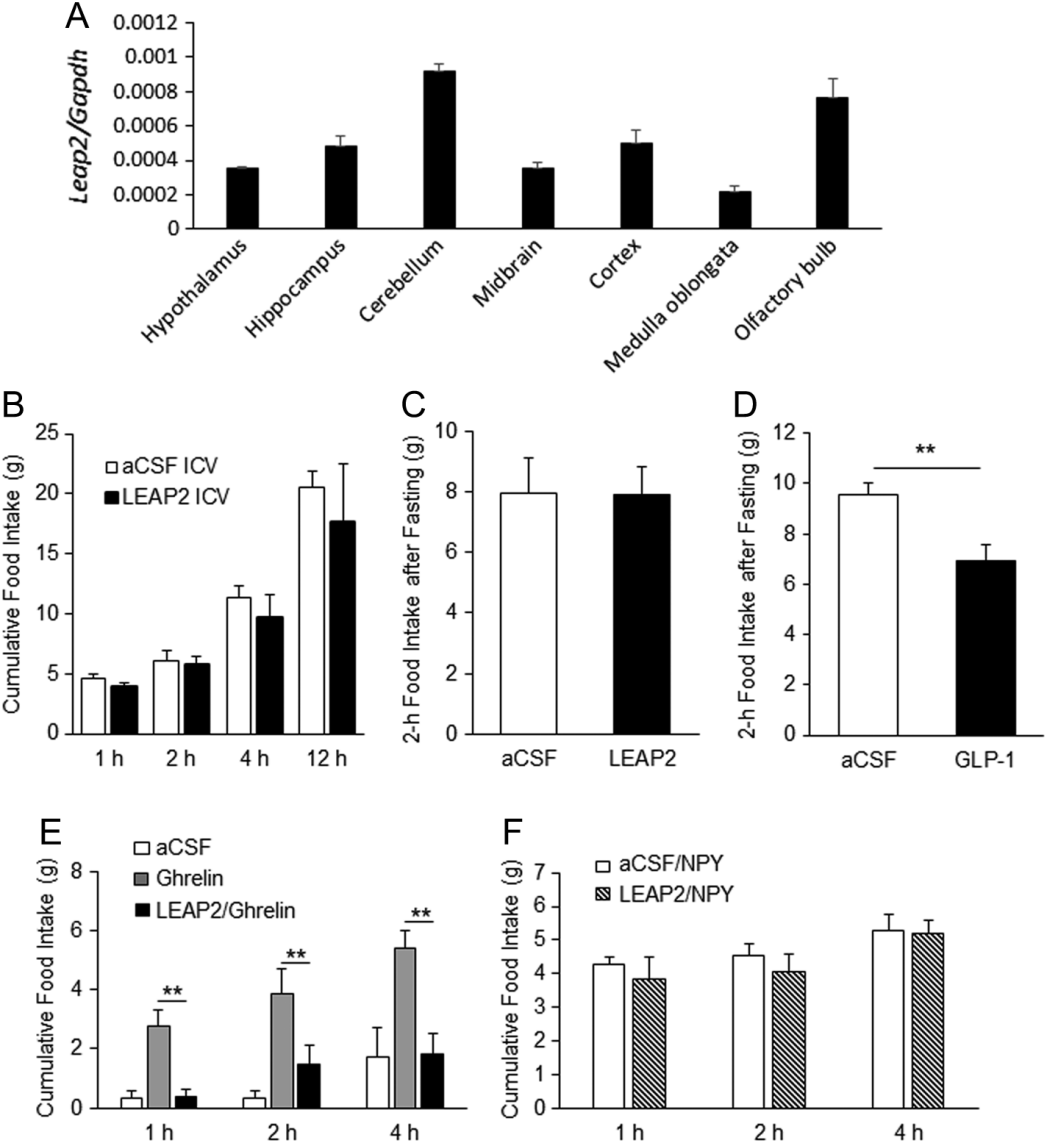
i.c.v. administration of LEAP2 suppressed ghrelin-induced feeding in rats. (A) *Leap2* expression in rat brain regions. i.c.v. administration of LEAP2 did not suppress (B) dark-phase food intake or (C) 14-h fasting-induced food intake. (D) GLP-1 suppressed fasting-induced feeding. LEAP2 suppressed (E) ghrelin-induced feeding but not (F) NPY-induced feeding (*n* = 4). Bars represent means ± s.e.m. **P < 0.01.

*Leap2* mRNA was expressed in various regions of rat brain (Fig. 4A). The activities of ghrelin are mediated through binding to the GHSR, which is expressed at high levels in the hypothalamic arcuate nucleus (ARC), dorsomedial hypothalamus (DMH), ventromedial hypothalamic nucleus (VMH), and lateral hypothalamic nucleus (LH) (Mitchell *et al.* 2001, Zigman *et al.* 2006). Hence, we investigated whether i.c.v. LEAP2 has any effects. i.c.v. administration of LEAP2 in the early dark-phase did not suppress dark-phase food intake (Fig. 4B). A similar effect was also observed in 14-h fasted rats (Fig. 4C), while central GLP-1 administration used as a positive control suppressed fasting-induced food intake (Fig. 4D).

Rats that received only ghrelin consumed significantly more food than control animals at 1, 2, and 4 h after single i.c.v. administration (Fig. 4E). This effect of ghrelin was significantly eliminated by co-administration of i.c.v. LEAP2 (Fig. 4E). To investigate whether LEAP2 had any effect on the inhibition of NPY-induced food intake, we administered either NPY alone or NPY + LEAP2. Co-administration of LEAP2 with NPY did not suppress NPY-induced food intake (Fig. 4F).

**Figure 5:**
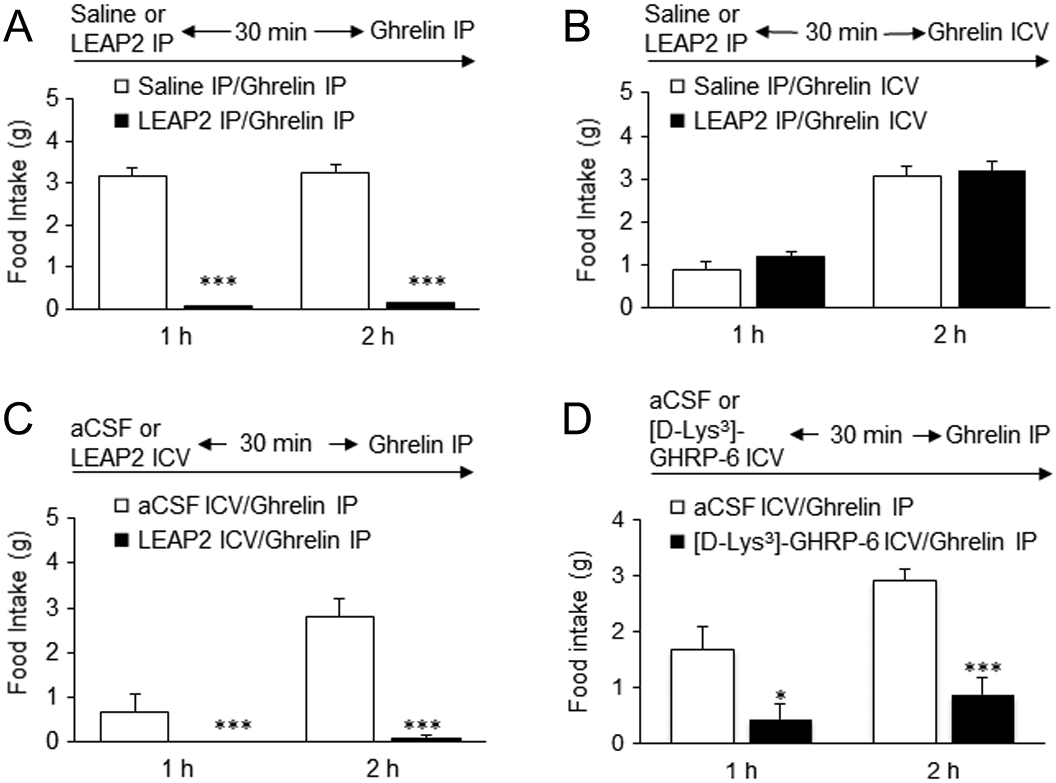
Effects of a combination of routes of administration of LEAP2 and ghrelin. i.p. LEAP2 administered 30 min before ghrelin administration suppressed the effect of i.p. ghrelin on feeding (A), but did not suppress i.c.v. ghrelin-induced feeding (B). i.p. ghrelin-induced feeding was suppressed by either i.c.v. LEAP2 (C) or i.c.v. [D-Lys^3^]-GHRP-6 administration 30 min beforehand (D) (*n* = 5). Bars represent means ± s.e.m. *P < 0.05; ***P < 0.001.

We investigated whether LEAP2 and ghrelin would be effective when administered via a combination of routes. To carry out the combination study, we first administered LEAP2 intraperitoneally; 30 min later, we introduced ghrelin through the i.p. route, and then measured 1-h and 2-h food intake. At both time points, LEAP2 significantly suppressed ghrelin-induced food intake (Fig. 5A). To determine whether peripheral LEAP2 could cancel the effect of central ghrelin, we performed another combination study in which we intraperitoneally administered LEAP2, and then centrally administered ghrelin 30 min later. LEAP2 did not abolish ghrelin-induced food intake (Fig. 5B). By contrast, i.c.v. administration of LEAP2 30 min beforehand abolished the effect of i.p. administration of ghrelin on feeding (Fig. 5C). i.c.v. administration of [D-Lys^3^]-GHRP-6, a GHSR antagonist, also suppressed the effect of i.p. injection of ghrelin on feeding (Fig. 5D).

### LEAP2 suppresses ghrelin-induced Fos expression in rats

**Figure 6:**
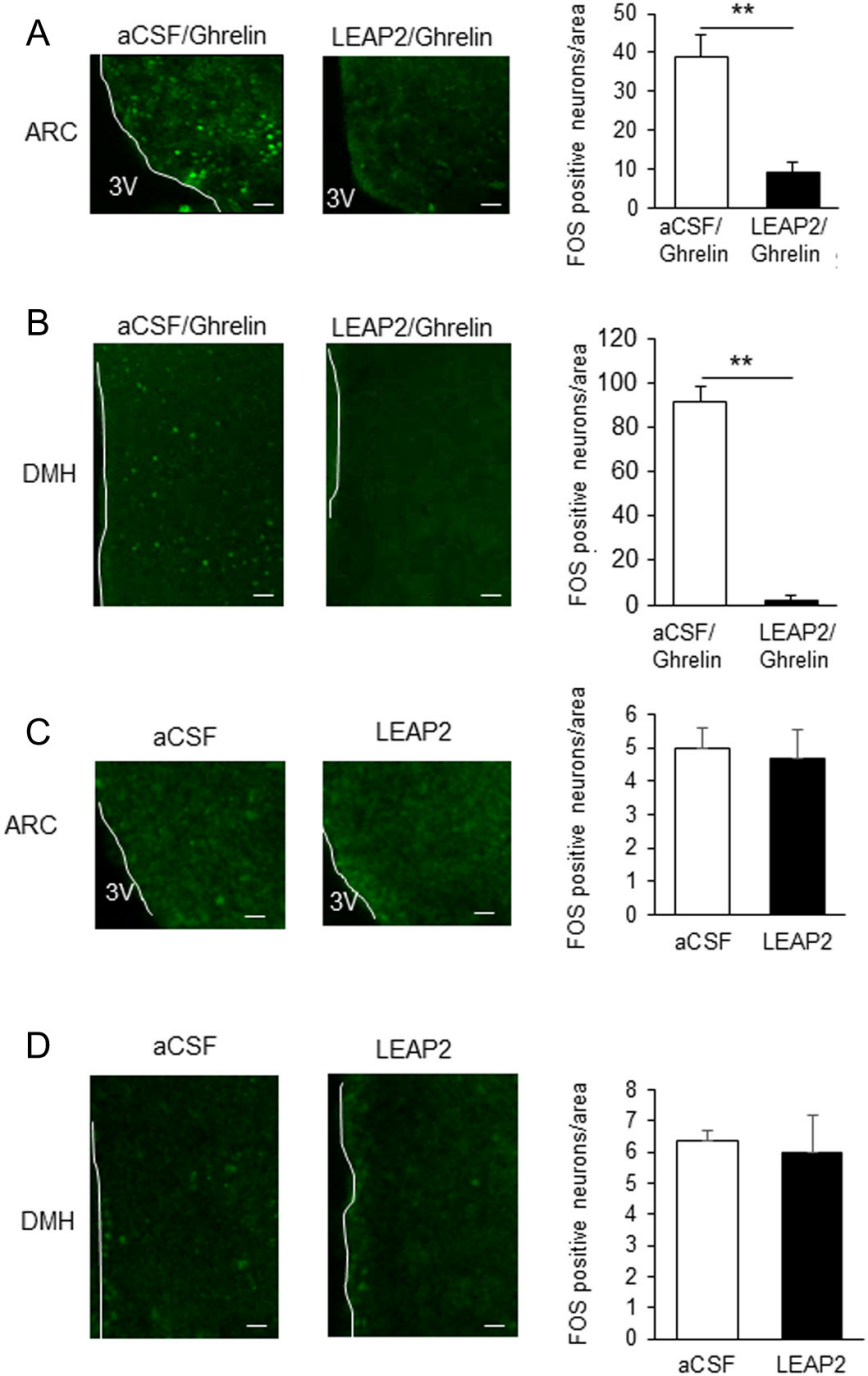
Fos expression in hypothalamic ARC and DMH. Fos expression was measured after i.c.v. administration of ghrelin with or without LEAP2 in ARC (A) and DMH (B) (*n* = 3). FOS expression was also measured after i.c.v. injection of vehicle aCSF or LEAP2 in ARC (C) and DMH (D). White line denotes the third ventricle (3V). Bars represent means ± s.e.m. **P < 0.01. Scale bars, 50 μm.

Ghrelin-induced Fos expression in ARC and DMH was significantly suppressed by i.c.v. LEAP2 when co-administered with ghrelin (Fig. 6A and B). i.c.v. LEAP2 also suppressed i.p. ghrelin-induced Fos expression in ARC (Supplementary Fig. 2). But single i.c.v. administration of LEAP2 did not show any significant Fos expression in ARC and DMH compared with vehicle aCSF injection (Fig. 6C and D).

### Antagonizing effects of LEAP2 on ghrelin-induced hyperglycemia, hypothermia and GH secretion

**Figure 7:**
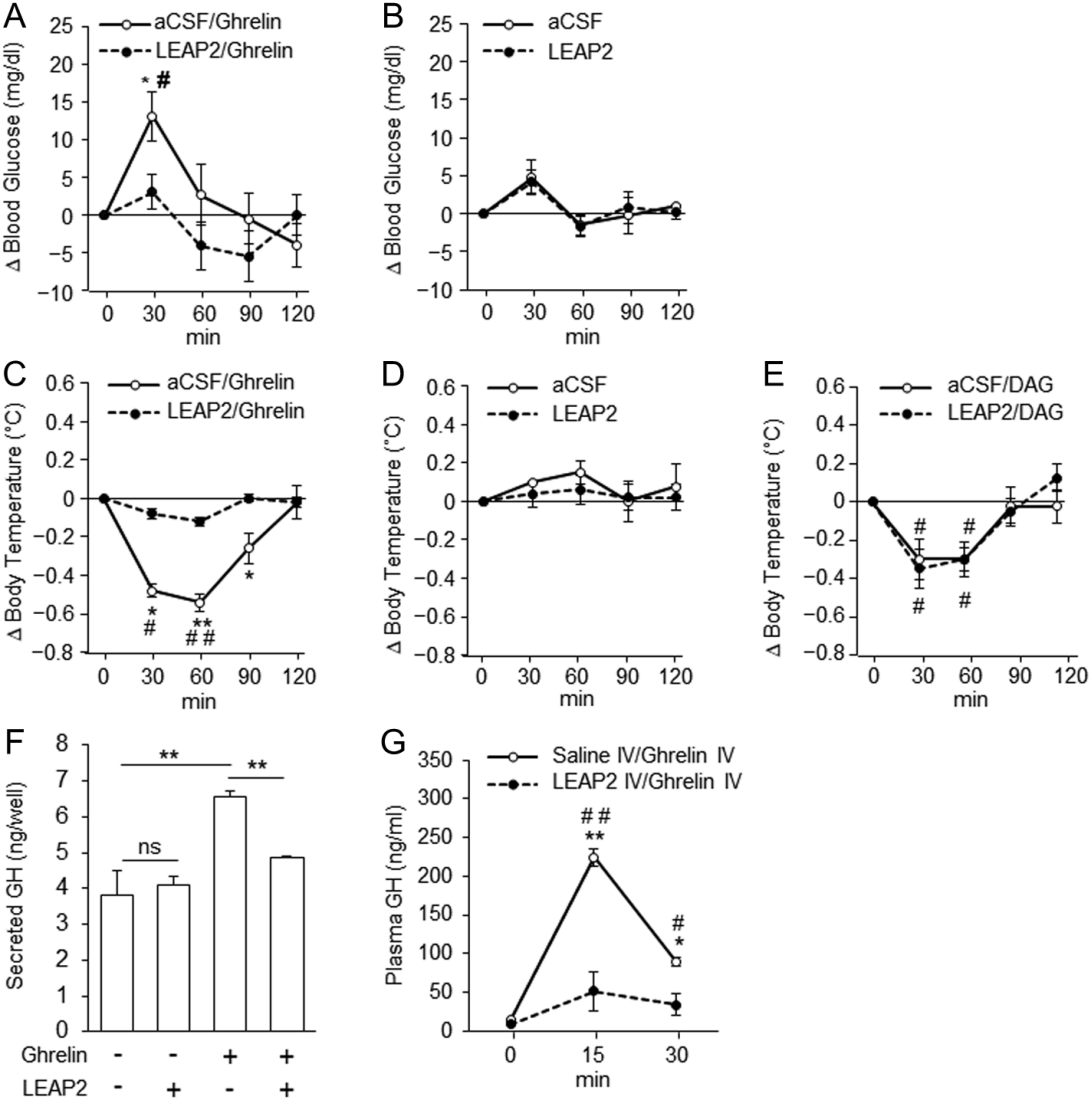
Ghrelin-specific antagonistic effect of LEAP2. Blood glucose elevation was abolished by co-administration of LEAP2 and ghrelin (A and B) (*n* = 5). LEAP2 antagonized the effect of ghrelin, but not des-acyl ghrelin, on body temperature (C, D and E) (*n* = 4). GH secretion from primary pituitary cells (F) (*n* = 4). GH secretion in rats (G) (*n* = 3). All data are presented as means ± s.e.m. *P < 0.05 and **P < 0.01, between two groups; ^#^*P* < 0.05 and ^##^*P* < 0.01 vs 0 min.

i.c.v. administration of ghrelin significantly elevated blood glucose concentration (Fig. 7A). This elevation was abolished by co-administration of i.c.v. LEAP2 (Fig. 7A). However, a single i.c.v. administration of LEAP2 had no effect on blood glucose (Fig. 7B). i.c.v. administration of ghrelin rapidly decreased body temperature, which reached a nadir 60 min after the injection. This reduction was abolished by co-administration of i.c.v. LEAP2 (Fig. 7C). i.c.v. administration of LEAP2 did not change body temperature (Fig. 7D). To investigate whether LEAP2 had any effect on des-acyl ghrelin-induced reduction in body temperature, we co-administered des-acyl ghrelin and LEAP2. LEAP2 had no effect on des-acyl ghrelin-induced body temperature reduction (Fig. 7E). Both pituitary primary culture and *in vivo* experiments demonstrated that LEAP2 abolished the effect of ghrelin on GH secretion (Fig. 7F and G).

## Discussion

In this study, we found that ghrelin regulated *Leap2* mRNA expression in the liver, and that central administration of LEAP2 abolished ghrelin-induced food intake, blood glucose elevation, and body temperature reduction. A previous study showed that peripheral administration of LEAP2 suppressed ghrelin-induced GH secretion and food intake in mice (Ge *et al.* 2018). Therefore, both central and peripheral LEAP2 antagonizes ghrelin functions.

Crosstalk between organs is important for maintenance of homeostasis. For example, feeding behavior is regulated by stomach-derived ghrelin which transfers the hunger signal to the hypothalamic arcuate nucleus via the vagus nerve (Yanagi *et al.* 2018), as well as by adipose tissue-derived protein leptin which also acts on the arcuate nucleus to suppress food intake (Friedman & Halaas 1998). In the present study, the plasma concentration of LEAP2 exhibited dynamics opposite to that of ghrelin; that is, LEAP2 decreased upon fasting and increased upon re-feeding, as did *Leap2* mRNA expression in the liver. Ghrelin administration to mice reduced the plasma concentration of LEAP2 and *Leap2* mRNA in the liver. These findings suggest that the concentration of plasma LEAP2 depends on the hepatic *Leap2* expression. Ghrelin administration did not suppress plasma LEAP2 or *Leap2* mRNA in the liver of GHSR-null mice which is further supported by the*in vitro* study in the primary hepatocyte of GHSR-null mice. Both plasma LEAP2 and *Leap2* mRNA expression in the liver of GHSR-null mice were not significantly differing than those of WT mice. The documented effects of ghrelin on feeding and GH release suggested that lack of ghrelin-GHSR signaling might affect the normal feeding and growth. Interestingly, GHSR-null mice did not show any significant difference in term of food intake, body weight and growth than those of their WT littermates (Sun *et al.* 2004, Zigman *et al.* 2005). Moreover, serum ghrelin, leptin and insulin levels were not significantly different between the two genotypes, both fed and fasting state (Sun *et al.* 2004). Combined with the result indicating no effect of des-acyl ghrelin on *Leap2* mRNA in Hepa 1-6 cells, ghrelin’s suppressive effect on *Leap2* expression was dependent on GHSR. We detected GHSR mRNA expression in both Hepa 1-6 and in the mouse liver. Although GHSR expression in the liver was low, it was enough for ghrelin to act on *Leap2* expression in the liver, as ghrelin was ineffective to suppress *Leap2* expression in the liver of GHSR-null mice, in both* in vitro* and *in vivo*. Chuang *et al.* reported that *Ghsr* expression depends on ambient glucose with high glucose suppress *Ghsr* expression (Chuang *et al.* 2011). The study implied condition dependent *Ghsr* expression in the liver which was profound under fasting state when blood glucose level became low. A previous study showed that ghrelin induced AMPK phosphorylation in rat hepatocytes (Ezquerro *et al.* 2016), and we here confirmed this finding in Hepa 1-6 cells. Phosphorylated AMPK suppresses the activity of sterol regulatory element-binding protein 1 (SREBP1), a key transcription factor involved in the regulation of lipid metabolism (Li *et al.* 2011). The binding consensus sequence of SREBP1 is present in the promoter region of *Leap2. Leap2* expression level may be regulated by the ghrelin–AMPK–SREBP1 pathway in the liver. This suggests that ghrelin affects feeding and energy homeostasis not only through the stomach–brain axis, but also through the stomach–liver axis.

Ghrelin binds to GHSR and promotes feeding by increasing expression of NPY and AgRP, hypothalamic downstream mediators of the GHSR pathway (Willesen *et al.* 1999, Nakazato *et al.* 2001). Ghrelin-induced feeding is canceled not only by inhibitors of GHSR, but also by neutralizing antibody against NPY and antagonists for NPY Y1 and Y5 receptors (Nakazato *et al.* 2001). We found that LEAP2 did not suppress NPY-induced feeding, suggesting that NPY is not a target of LEAP2. Both acyl ghrelin and des-acyl ghrelin decrease body temperature in rats (Lawrence *et al.* 2002, Inoue *et al.* 2013), whereas des-acyl ghrelin has been thought to have specific receptor other than GHSR (Sato *et al.* 2006, Lear *et al.* 2010). Consistent with this, LEAP2 did not suppress des-acyl ghrelin-induced reduction in body temperature. Together, these results imply that LEAP2 targets GHSR.

Recently published two articles on LEAP2 research demonstrated that entire sequence of LEAP2 peptide was not necessary to bind to its target receptor GHSR (M’Kadmi *et al.* 2019, Wang *et al.* 2019). Indeed, N-terminal region of the LEAP2 peptide exhibited inverse agonist activity toward the GHSR and was able to inhibit subcutaneous ghrelin-induced food intake in mice (M’Kadmi *et al.* 2019). In contrast, Ge *et al.* reported LEAP2 as a noncompetitive antagonist to the GHSR (Ge *et al.* 2018). Interestingly, another form of LEAP2 peptide (44-77) lacking N-terminal region was found in the circulation which did not display antimicrobial activity. But two disulfide bonds are necessary for the stabilization of the LEAP2 peptide (Krause *et al.* 2003). This implies that N-terminal region of the LEAP2 peptide may facilitate for the binding and core region containing two disulfide bonds are crucial for the stability of the peptide. However, further study is needed to investigate the nature of action of N-terminal LEAP2 toward GHSR. It is also necessary to investigate whether this short fragment of LEAP2 peptide is stable at *in vivo* condition or whether it exhibits inhibitory effects of ghrelin action besides feeding behavior, for example, on ghrelin-induced GH release.

Ghrelin acts directly on the pituitary gland to induce GH secretion (Kojima *et al.* 1999). We previously reported that GHSR mRNA expressed in vagal nodose ganglion and binding of radiolabeled ghrelin in the vagus nerve (Date *et al.* 2002) Bilateral subdiaphragmatic vagotomy, selective gastric branch vagotomy, or perivagal capsaicin injection blocked the feeding stimulatory effects of i.v. ghrelin in rats (Date *et al.* 2002) and bilateral subdiaphragmatic vagotomy blocked the i.p. ghrelin-induced feeding in mice (Asakawa *et al.* 2001). Moreover, intravenous ghrelin infusion failed to increase feeding in human after bilateral subdiaphragmatic vagotomy and lower esophageal or gastric surgery (Ie Roux *et al.* 2005). Consistent with the vagal involvement in the feeding stimulatory effects of ghrelin, ghrelin also decreased vagal afferent activity in rats and an intact vagal afferent nerve activity is required to induce i.v. but not i.c.v. ghrelin-induced Fos expression in the ARC (Date *et al.* 2002). i.c.v. ghrelin induces the Fos expression in various brain regions but peripheral ghrelin induces the hypothalamic ARC Fos expression only (Hewson *et al.* 2000, Nakazato *et al.* 2001, Date *et al.* 2002, Cabral *et al.* 2014). Thus, a divergent pattern of Fos expression is observed by central or peripheral ghrelin administration. However, GHSR expression in vagal nodose ganglion is necessary to exhibit the ghrelin-induced food intake (Okada *et al.* 2018). On the other hand, ghrelin crosses the blood–brain barrier from blood-to-brain and from brain-to-blood in a saturated transport system (Banks *et al.* 2002, Rhea *et al.* 2018, Banks 2019). Ghrelin in CSF is accessible to the most areas of the brain where GHSR is expressed abundantly (Cabral *et al.* 2013) and centrally administered GHSR antagonist abolished the peripherally ghrelin-induced feeding (Asakawa *et al.* 2003). Together, these reports indicate that both vagal nerve innervation and direct activation of brain GHSR by peripheral ghrelin are required to display ghrelin-induced food intake. It remains unclear how LEAP2 expression is regulated in the brain and whether LEAP2 can cross the blood–brain barrier. We here showed that both i.c.v. LEAP2 and i.c.v. [D-Lys^3^]-GHRP-6 inhibited i.p. ghrelin-induced feeding and Fos expression in the ARC. These results suggest that GHSR activation in the brain is required for ghrelin-induced feeding when administered peripherally. LEAP2 administration to isolated pituitary glands suppressed ghrelin-induced GH secretion, whereas peripheral LEAP2 did not impact i.c.v. ghrelin-induced food intake. In addition, peripheral administration of a neutralizing antibody against LEAP2 increased GH secretion (Ge *et al.* 2018). These findings suggest that LEAP2 in the brain acts on pituitary to suppress GH secretion. The regulation of LEAP2 expression in the brain is important to understand central ghrelin functions.

In conclusion, our results demonstrate that ghrelin and LEAP2 cooperate to regulate feeding and energy homeostasis through two mechanisms: crosstalk between stomach and liver, and direct inhibition of peripheral and central GHSR by LEAP2. LEAP2 agonism represents a possible therapeutic target for obesity and diabetes, and LEAP2 antagonism for anorexia and cancer cachexia. Further study is needed to investigate the efficacy and safety of LEAP2 as a treatment for these diseases.

## Supplementary Material

Supplementary Figure 1: Tissue distribution of Leap2 mRNA levels. (A) Three-dimensional digital PCR data showed that Leap2 was highly expressed in rat liver (n = 4), consistent with results determined by qRT-PCR (Fig. 1). (B) In mice, Leap2 mRNA expression determined by qRT-PCR was most abundant in jejunum (n = 5).

Supplementary Figure 2: ICV LEAP2 suppressed IP ghrelin-induced Fos expression. Schematic illustration of the Fos experiment (A). Fos expression in the hypothalamic ARC either ICV aCSF (B) or LEAP2 (C) injection followed by IP ghrelin injection (n = 3). Bars represent means ± SEM (D). **P < 0.01, White line denotes the third ventricle (3V). Scale bars, 50 μm.

Supplemental Table 1: Quantification of LEAP2 ELISA. A linear relationship between the quantity of LEAP2 and the volume of plasma was noted. The synthetic LEAP2 added to the plasma sample was recovered by this ELISA system.

## Declaration of interest

The authors declare that they have no conflict of interest that could be perceived as prejudicing the impartiality of the research reported.

## Funding

This research was supported by JSPS KAKENHI (No. 16H05333) and AMED-CREST (No. JP18gm0610016) to M N.

## Author contribution statement

Y M, H S, and M N designed the experiments; M N I, Y M, R T, W Z and K M performed the experiments; and M N I, Y M, R T, W Z and K M analyzed the data. All authors prepared and approved the final version of the manuscript.
