# Supplementary figures and images for: Liver-expressed antimicrobial peptide 2 antagonizes the effect of ghrelin in rodents
Source: J Endocrinol. 2019 Sep 19;244(1):13–23. doi: 10.1530/JOE-19-0102 (PMC6839046; doi:10.1530/JOE-19-0102)

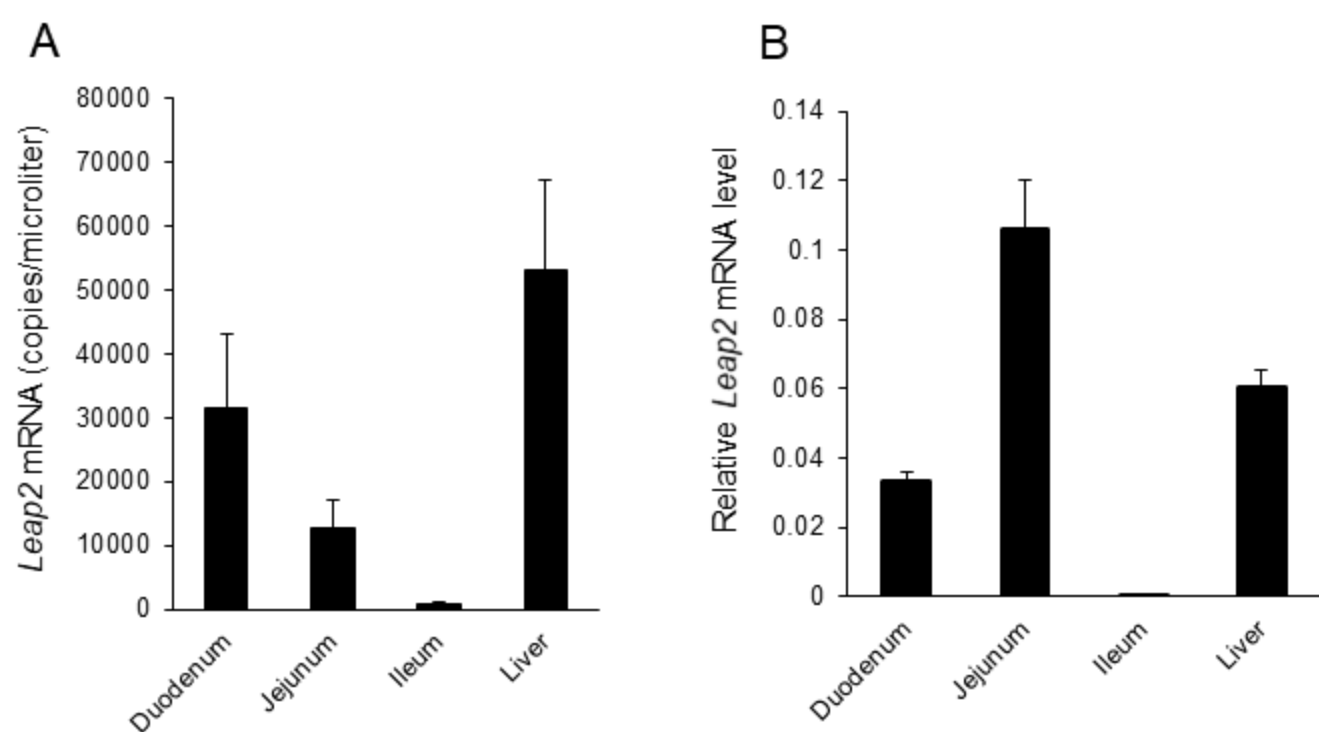

Supplementary Fig. 1

Supplement: Supplementary Figure 1: Tissue distribution of Leap2 mRNA levels. (A) Three-dimensional digital PCR data showed that Leap2 was highly expressed in rat liver (n = 4), consistent with results determined by qRT-PCR (Fig. 1). (B) In mice, Leap2 mRNA expression determined by qRT-PCR was most abundant in  [file supplementary_figure_1.pdf]

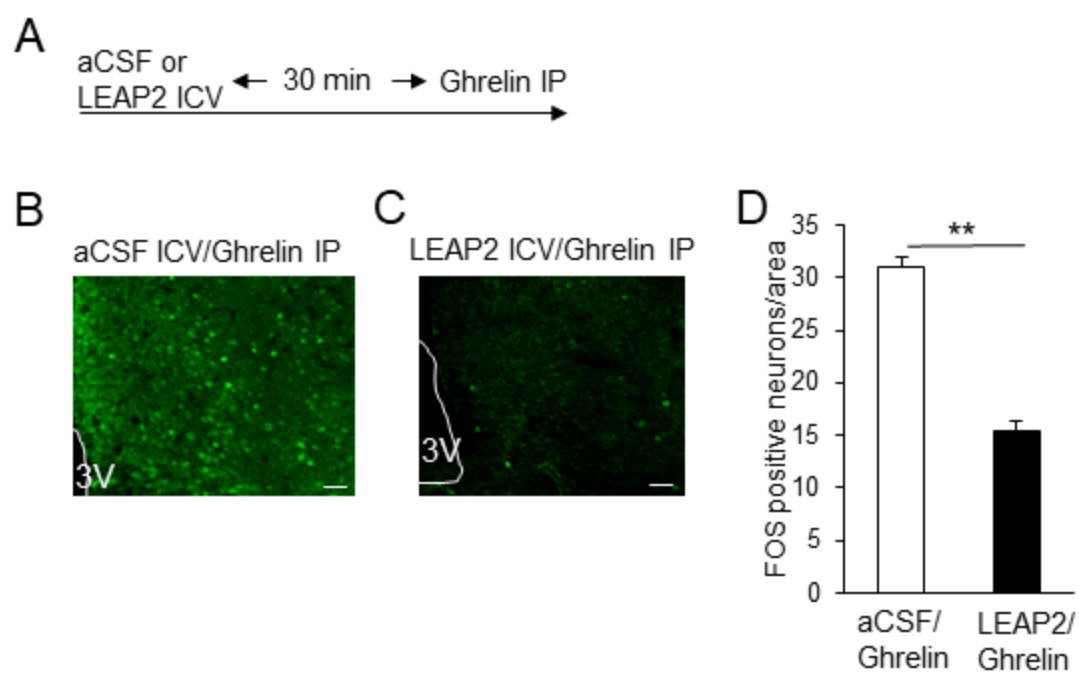

Supplementary Fig. 2

Supplement: Supplementary Figure 2: ICV LEAP2 suppressed IP ghrelin-induced Fos expression. Schematic illustration of the Fos experiment (A). Fos expression in the hypothalamic ARC either ICV aCSF (B) or LEAP2 (C) injection followed by IP ghrelin injection (n = 3). Bars represent means ± SEM (D). **P < 0.01, Wh [file supplementary_figure_2.pdf]
