# Supplementary material for: Liver-expressed antimicrobial peptide 2 antagonizes the effect of ghrelin in rodents
Source: J Endocrinol. 2019 Sep 19;244(1):13–23. doi: 10.1530/JOE-19-0102 (PMC6839046; doi:10.1530/JOE-19-0102)
Supplement: Supplemental Table 1: Quantification of LEAP2 ELISA. A linear relationship between the quantity of LEAP2 and the volume of plasma was noted. The synthetic LEAP2 added to the plasma sample was recovered by this ELISA system. [file supplementary_table_1.pdf]

		Rat-1	Rat-2
Added LEAP2 peptide (ng)	Plasma (µl)	LEAP2 (ng)	LEAP2 (ng)
0	80	17.1	21.9
0	40	9.5	11.6
0	20	4.8	6.1
7.5	20	12.2	13.9

Supplemental Table. 1
